# Suicidality and Personality Pathology in Adolescence: A Systematic Review

**DOI:** 10.1007/s10578-021-01239-x

**Published:** 2021-09-15

**Authors:** Marta Moselli, Maria Pia Casini, Camilla Frattini, Riccardo Williams

**Affiliations:** 1 Department of Clinical and Dynamic Psychology, Sapienza University of Rome, Rome, Italy; 2 Child Neuropsychiatry Unit, Department of Neuroscience, I.R.C.C.S. Children Hospital Bambino Gesù, Rome, Italy

**Keywords:** Adolescence, Suicidal conducts, Suicidal risk, Personality pathology, Review

## Abstract

This work presents a review of research papers examining the role of emerging personality pathology in suicidal ideation and behaviours in adolescence. Initially, 226 studies were selected in line with PRISMA guidelines, and 33 articles were finally included in this review. The data show percentages of any personality disorder diagnosis ranging from 19.5 to 22.8% in suicide attempters, while in autopsy studies, the rate of personality disorder diagnosis varied between 29.6 and 42.1%. The overwhelming majority of the studies focus on the role of borderline personality disorder (BPD) in suicidal behaviours, also highlighting its predictive role at a longitudinal level. Furthermore, the literature review shows that personality traits supposed to underlie BPD, such as affective instability, impulsivity and identity diffusion, have specific predictive links with suicidal conduct. Other personality pathology dimensions, such as aggressiveness, sadism and perfectionism that are associated with other personality disorders, namely, antisocial and narcissistic personality disorders, have also shown a significant mediating role for suicidal risk. Overall, these results seem to parallel the role of personality pathology in predicting suicide in adulthood and point to the relevance of assessing the presence of emerging patterns of personality disorders for the clinical management of suicidal risk in adolescence.

## Introduction

Suicide is considered a public health problem worldwide, and the World Health Organization has declared its reduction as a primary objective at a global level [1]. Suicide is the second-leading cause of death after road accidents in subjects aged between 12 and 34 years [2, 3], and the maximum peak incidence of suicidal behaviours and suicidal ideation is indeed observed during adolescence [4]. The literature on suicidality both in adulthood and adolescence has shown the interaction of an array of potential risk factors in determining the shift from suicidal ideation to actual suicidal conduct. Consequently, diverse risk factors have been investigated, although the specific factors that may reliably differentiate high- and low-risk patients have not yet been fully defined. Among the risk factors for suicide, psychiatric disorders have been considered prominent. The psychiatric disorder most frequently associated with suicidal behaviours—both in adulthood and in adolescence—is major depression, whether in its unipolar form or as an episode in a bipolar disorder [5, 6]. It has also been shown that personality disorders (PDs) represent a severe risk factor for suicidality in adulthood [7, 8]. In fact, a meta-analysis of 27 psychological autopsy studies reported that 16% of adult suicide victims had been diagnosed with a PD [9]. Moreover, studies have shown that the younger the onset of suicidal behaviours, the higher the likelihood of a co-occurrence of a diagnosis of cluster B PDs [10–13]. In fact, the diagnosis of PDs in adolescence has been a controversial matter: recent innovations proposed by both the alternative model for PD in the Diagnostic and Statistical Manual of Mental Disorders, 5th edition (DSM-5) (“Materials and Methods” section) and International Classification of Diseases, 11th revision (ICD-11) reflect increasing attention towards the assessment of personality pathology during this developmental age [13]. In fact, the DSM-5 allows the diagnosis of PD in adolescence if a greater than 1-year pattern of immature personality development with disturbances in different specific domains (depending on the type of PD) is observed [14]. In fact, diagnoses of PDs in adolescence regularly show both concurrent and predictive stability with respect to measures of functional impairment and psychopathology in many domains of adjustment throughout the life span [13, 15]. Furthermore, over time, adolescents with significant functional impairments often shift from one diagnostic category to another [16]. Overall, in both the developmental and adulthood literatures, this instability and discontinuity of PD diagnoses—‘heterotypic continuity’—have led to a possible reformulation of PDs as clinical variants of underlying basic psychobiological dimensions that present robust developmental stability and significant genetic loading [17–21]. Following this indication, the DSM-5 has introduced a dimensional-categorical PD hybrid model with the aims of reducing comorbidity and improving the validity and stability of diagnoses [22]. The presence of PDs in adolescence, especially those of clusters B and C, has been associated with a high risk of suicidal ideation and suicide attempts, including at a prospective level in early adulthood [13]. Adolescents suffering from a PD are at greater risk of developing many of the problems associated with suicidal behaviour, and although the implications for the risks of attempted and completed suicide may differ, personality pathology is implicated in both conditions [23]. Although studies investigating the presence of PDs in adolescent populations with suicidal behaviours have shown a significant prevalence of PDs in these samples [24–26], to date, there is no systematic review examining suicidality and personality pathology in adolescence. In this paper, we propose to provide such a review by employing both the categorical approach based on diagnoses of PDs in adolescence and the dimensional approach investigating those personality traits that, according to empirical literature, are more consistently related to personality pathology during the lifespan.

Given the impact of suicidal conduct in adolescence and the relevance of personality pathology for suicidality in adulthood, this review has the following aims:

1. To assess the prevalence and the association of any DSM and ICD personality disorder diagnosis, traits (number of criteria met for any disorder) or dimensions with suicidal ideations and conduct in adolescence in both clinical and community populations.
2. To assess the prevalence and the association of DSM- and ICD-specific personality disorder diagnosis, traits (number of criteria met for any disorder) or dimensions with suicidal ideations and conduct in adolescence in both clinical and community populations.
3. To assess the association of personality pathological dimensions/traits associated with DSM- or ICD-specific personality disorders with suicidal ideations and conduct in adolescence in both clinical and community populations.

## Materials and Methods

### Literature Search

A systematic search of the PubMed/MEDLINE research literature databases was carried out to identify relevant, peer-reviewed articles on personality disorders/traits/dimensions in adolescents with suicidal behaviours. The search syntax was based on combinations of the following terms: personality AND (disorder OR pathology OR traits) AND (suicide OR suicide attempt) AND (adolesc* OR child*) AND [(“1980/01/01”[PDat]: “2020/01/01”[PDat])]. The search was undertaken from 1 March 2017 to 30 January 2020. We also examined reports as well as the abstracts from recent psychiatric research conferences. This systematic review was performed in accordance with Preferred Reporting Items for Systematic Reviews and Meta-Analyses (PRISMA) guidelines [27].

### Study Selection

We included studies investigating the prevalence of PDs or trait diagnoses and personality dimensions commonly associated with PDs, e.g., those observed in the suicidal population, both in clinical and community samples. We also included studies examining the prevalence of suicidality in both clinical and community samples screened for assessments of personality disorders, traits and dimensions in association with adolescents’ suicidal ideation or behaviour. All studies included in this review considered clinical or community populations with subjects aged between 13 and 18 years old. Regarding the definition of suicidality, we referred to the Columbia Classification Algorithm of Suicide Assessment (Posner, 2007) and included studies dealing with suicidal ideation, suicidal intention, suicidal plans and suicidal behaviours. Studies dealing with self-harming behaviours that did not clearly facilitate the identification of subjects with the idea or intention to die were excluded. Regarding PD diagnoses, we included studies published since 1980, the year that the DSM-III was published, as it introduced the categories of PDs as we know them today. No study focusing on an alternative model of DSM-5 PDs with respect to suicidality was found. The following contributions were excluded from our review: reviews, meta-analyses, editorials, letters and comments, case reports and studies not based on standardized criteria. Two experienced investigators (C.F. and M.M.) reviewed the titles and abstracts of the retrieved articles and applied the study’s inclusion and exclusion criteria. These investigators then independently reviewed the full-text articles to confirm the studies’ eligibility for inclusion.

### Data Extraction

For each included study, two authors (C.F. and M.M.) collected basic information (authors, reference citation, year, country) and study characteristics, including research design, diagnostic methods, diagnoses and rates of PDs, other diagnoses and comorbidities, participant counts, proportions by sex, age range and mean, rates of suicide attempts, definition of suicide attempt, methods of evaluating attempts and follow-up.

## Results

**Fig. 1:**
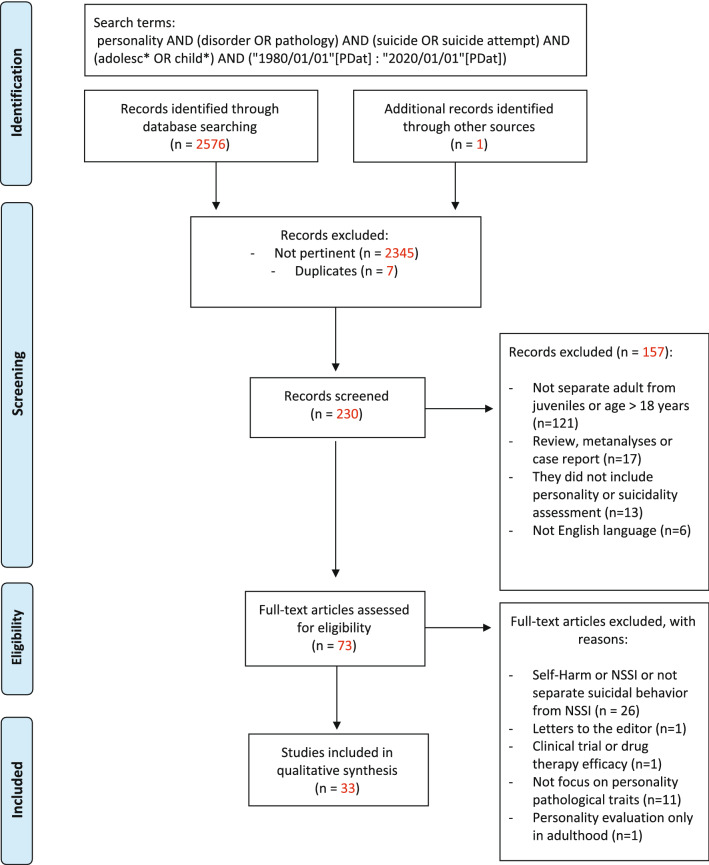
Preferred Reporting Items for Systematic Reviews and Meta-Analyses (PRISMA). Reproduced with permission. [27] © 2009 Moher et al. From: Moher D, Liberati A, Tetzlaff J, Altman DG, The PRISMA Group (2009). Preferred Reporting Items for Systematic Reviews and Meta-Analyses: The PRISMA Statement. PLoS Med 6(7): e1000097. 10.1371/journal.pmed1000097

To achieve a complete and clear-cut picture, the results regarding each objective will be divided into cross-sectional and longitudinal sections. The literature search generated 2576 potential records, plus one from the grey literature of initially identified reports, resulting in 230 records to be screened. Of these, 157 were excluded for the following reasons, as shown in Fig. 1: they included adults or did not separate adults from juveniles (n = 121); they were reviews, meta-analyses or case report articles (n = 17); they did not include personality or suicidality assessments (n = 13); or they did not use the English language (n = 6). Among the 73 selected research studies, 40 full-text articles were excluded because they did not separate suicidal ideation or behaviour from non-suicidal self-injury (NSSI) or self-harm and instead either examined only self-harming behaviour, were letters to editors or clinical trial or drug therapy efficacy studies, were focused on non-pathological personality traits, or assessed personality pathology only in adulthood (Fig. 1). The remaining 33 articles had different focuses, analysing the presence of PDs overall, specific personality categories and pathological personality traits in samples with suicidal ideation and/or suicide attempts and completed suicides.

### Overall Personality Disorders

#### Cross-sectional Studies

Four studies investigated the relationship between suicidality and the presence of PDs overall. With respect to actual suicidal behaviour, the presence of any PD was found in 19.5–22.8% of adolescents who attempted suicide [28, 29] and in 29.6–42.1% of adolescent suicide victims [30, 31], with higher percentages obtained when cumulating PDs and traits as diagnosed using the ICD-10 criteria (Table 1). Tairi et al. found a subgroup at greater risk in an adolescent attempter population characterized by a PD [32].

**Table 1:** Personality disorders as a risk factor for suicidal ideation and conducts, prevalence and notable findings

Studies^a^	Study type	Sample type	Personality assessment	Findings
Cross et al. [23]	Cross sectional	Clinical attempters n =267	(1) Shedler-Westen Assesment Procedure (SWAP-II-A); (2) Axis II Personality Checklist	The narcissistic subtype (n = 12) is positively correlated with NPD (r = 0.17), as well as with school functioning (r = 0.27). This subtype is negatively correlated with CD (r = j0.16), SUD (r = j0.14), and externalizing pathology (r = j0.16). Although similar to the high functioning subtype, this subtype is characterized by less internalization and more by narcissism
Groholt and Ekeberg [27]	Prevalence follow-up = 9 years	Clinical attempters n T1 = 87 [PD 19.5%] n T2 (adulthood) = 71. [PD 34%]	Mini International Neuropsychiatric Interview (MINI)	The stability of diagnoses was moderate
Villar et al. [28]	Prevalence follow-up = 6 months	Clinical attempters n = 417 (PD or PD traits 22.8%)	Diagnosis made by DMS-IV	A statistically significant model χ^2^(3, N = 417) = 18.610; p < 0.001; Nagelkerke R^2^ = 0.096 including the following factors was obtained: current diagnosis of personality disorder/maladaptive personality OR = 0.806, p = 0.028, 95% CI [1.091, 4.595], personal history of self-injury OR = 0.728, p = 0.043, 95% CI [1.023, 4.192], and family history of psychopathological diagnosis OR = 0.925, p = 0.021, 95% CI [1.151, 5.530]
Houston et al. [29]	Psychological autopsy	Victims n = 27 (PD 29.6%; Dissocial 14.8%; Anankastic 7.4%; Anxious 3.7%; Paranoid 3.7%)	Personality Assessment Schedule (PAS) [ICD - 10]	Psychiatric disorders were diagnosed in 19 (70.4%) subjects. These were most commonly depressive disorders (55.5%). Very few individuals were receiving treatment for their disorders. Personality disorders were present in 29.6% of subjects and disorders or personality trait accentuation in 55.6%. Comorbidity of psychiatric disorders was found in a third of subjects
Portzky et al. [30]	Psychological autopsy	32 informants for 19 suicide cases [PD 42.1%; Paranoid 5.3%; Emotionally unstable (borderline) 5.3%; Anankastic 5.3%; Anxious 5.3%; Dependent 21.1%]	Personality Assessment Schedule (PAS) [ICD - 10]	All adolescents were suffering from one or more mental disorder(s) at the time of their death, and almost half of them were diagnosed with personality disorders. Adjustment disorders were diagnosed in one fifth of the sample, which appears to be relevant in view of the multiple life events and other psychosocial problems which adolescents were facing shortly before death
Tairi et al. [31]	Cross sectional	Clinical attempters n = 182 (PD 19.2%)	Diagnosis made by DMS-IV	We observed two distinct classes, specifically in the probability of mood disorders, substance use disorders, abandonment/neglect, and displaying traits of personality disorders. While most of the adolescents who attempted suicide showed a low probability of these parameters (71.7%), about a third of the sample (28.3%) showed a much more severe clinical profile. Analyses of pertinent contextual and risk factors indicated that those with a more severe clinical profile tend to come from overall more dysfunctional family systems, have more problems in school, and have made a previous attempt
Ayodeji et al. [32]	Prevalence follow-up = 6/12 months	Clinical sample n = 357	Structured Clinical Interview for DSM-IV Axis II disorders (SCID II)	In univariate analysis, the presence of personality disorder was associated with greater suicidal ideation (SIQ; OR = 1.01, p = 0.001, 95% CI [1.01–1.02]). In multivariate analysis, the association with suicidal ideation (SIQ; OR = 1.01, p = 0.005, 95% CI [1.00–1.01]) remained
Kuba et al. [33]	Clinical Trial	Clinical outpatien with suicidal related events n = 70 (BPD 56.3%)	Diagnosis made by DMS-IV	The proportion of SRE decreased from 47.1 to 22.9% after the treatment. Subjects with persistent risks of SRE were significantly characterized by female sex (p < 0.05), psychotic features (p < 0.001), borderline personality disorder (p < 0.01), previous SRE (p < 0.001), and such baseline psychopathology as anhedonia (p < 0.005), irritability (p < 0.005) and hopelessness (p < 0.001). Discriminant analysis showed that baseline severity of SRE, borderline personality disorder and psychotic features were closely associated with SRE during antidepressant therapy
Greenfield et al. [34]	Prevalence follow-up = 6 months	Clinical outpatients suicidal group n = 77 (BPD 90.9%)	Abbreviated Diagnostic Interview for Borderlines (Ab-DIB)	BPD, previous suicide attempt(s), drug use and female gender were associated with subsequent suicidality (BPD Suicidal 90.9%, BPD non suicidal 72.6%; odd ratio 3.8, p = 0.002 [CI 1.6–8.7]). BPD results ad indipendent predictor of suicidality (odd ratio= 2.40, p = 0.05, 95% CI [0.99–5.79])
Greenfield et al. [35]	Prevalence follow-up = 4 years	Clinical attempters baseline n = 286; Follow-up = 229 (n = 204 with personality data at both point) (BPD 76%)	Abbreviated Diagnostic Interview for Borderlines (Ab-DIB) - self report	Intra-class correlation analyses indicate that overall BPD diagnosis presented considerable stability (ICC = 0.603; 95 % CI [0.40–0.78]; Odds ratio = 8.02, 95 % CI [4.01–16.84]). Only 17 (7.8 %) of 219 patients remained suicidal (scored greater than 1 on the SSBS) at follow-up, 16 (94.1 %) of whom met BPD criteria
Fritsch et al. [36]	Cross sectional	Clinical attempters n = 35 (BPD 6%); clinical no attempts n = 102	1) Millon Adolescent Personality Inventory (MAPI); 2) revised Diagnostic Interview for Borderlines (DIB-r).	No distinctive personality characteristics or symptoms of personality disorders were found. However, affective distress seemed to be the most prominent feature in the presentation of these adolescents. Additionally, high scores on the HSC were associated with elevated scores on the Personality Style scales of the MAPI and higher (more dysfunctional) scores on Affect Regulation on the DIB
Kato et al. [37]	Cross sectional	Clinical inpatient n = 79 (BPD 12.6%)	Diagnosis made by DMS-IV	The study compare suicide attempts in BPD and non-BPD patients among adolescents in Japanese emergency rooms. The suicide attempt history was significantly higher in the BPD group (90% vs 13.4%, p = 0.002). The proportion of patients with mood disorders was significantly higher (p = 0.009) in the BPD (70%) than in the non-BPD group (26.1%). A number of adolescent BPD patients may also have mood disorders at the time of attempted suicide, which suggests that treatment of mood disorders may help prevent recurrent suicide attempts in adolescent BPD patients
Yen et al. [38]	Prevalence follow-up = 6 months	Clinical inpatient Baseline n = 119; Follow up n = 104; Suicidal n = 37 (BPD 48.6%)	Childhood Interview for Borderline Personality Disorder (CI-BPD)	After removing the self-injurious behaviors criterion to mitigate the possibility that the item reflected a suicide attempt, the sum of the remaining eight BPD criteria operationalized continuously was a significant predictor of time to suicide event (Wald χ^2^ = 6.44, OR = 1.22, p = 0.01)
Selby and Yen [39]	Prevalence follow-up = 6 months	Clinical inpatients n = 119 (BPD 40%)	Childhood Interview for Borderline Personality Disorders (CI-BPD)	There was a significant time interaction with BPD diagnosis, such that the BPD group showed larger linear decreases in suicidal ideation over the duration of the follow-up, F(1,77) = 5.128, p < 0.05, gp^2^ = 0.062, than those without a BPD diagnosis. Despite the potential curve the quadratic time interaction with BPD diagnosis was not significant, F(1,77) = 2.59, p > 0.05, indicating that the rate of decrease in suicidal ideation for the BPD group was relatively constant throughout follow-up
Horesh et al. [40]	Cross sectional	Clinical attempters n = 65 (BPD group n = 33; No BPD group n = 32)	Diagnostic Interview for Borderlines (DIB-R)	There were no significant differences in impulsiveness for the MDD suicidal group versus the MDD nonsuicidal group, but the suicidal BPD adolescents were significantly more impulsive than the nonsuicidal BPD adolescents. Only anger out significantly differentiated suicidal from nonsuicidal adolescents (F(1,62) 4.64, p 0.05), with anger out higher (mean, 15.15; SD, 3.90) in the nonsuicidal group than in the suicidal group (mean, 13.00; SD, 4.70). The BPD group had more outward anger than the MDD group (mean, 15.58; SD, 4.15; versus mean, 12.30; SD, 3.95; F(1,62) 13.68, p 0.01). On suicide intent (SIS), the suicidal subjects with MDD had significantly higher intent scores than the suicidal adolescents with BPD (MDD: mean, 17.5; SD, 6.45; versus BPD: mean, 12.2; SD, 4.2; t(31) 2.79; p 0.01)
Goodman et al. [41]	Cross sectional	Clinical sample (inpatient + outpatient) (adolescent BPD group n = 104, attempters 76%; adult BPD group n = 290, attempters 79.3%)	(1) Structured Clinical Interview for DSM-IV Childhood Diagnoses (KID-SCID); (2) Diagnostic Interview for Borderlines (DIB-R); (3) Childhood Interview for DSM-IV Borderline Personality Disorder (CI-BPD)	There is substantial overlap between adolescent and adult BPD in the rates of suicide attempts: the only significant difference in the number of attempts s was ‘five or more’ (adult BPD group 32% vs adolescent BPD group 15.4%; OR= 0.37, p < 0.001, 95% CI [0.20–0.68], Z = 3.21). Adolescents with BPD were significantly younger at the time of their first suicide attempt (M = 14.0 years, SD = 2.5) than adults with BPD (M = 19.0 years, SD = 6.8), t(307) = 6.49, p < 0.001
Knafo et al. [42]	Cross sectional	Clinical inpatient attempters n = 162 (BPD 62%)	Abbreviated Diagnostic Interview for Borderlines (Ab-DIB) - self report	Compared with adolescents without BPD, adolescents with BPD presented more severe suicidal ideation (BPD 26%, no BPD 5%; p < 0.001), behaviour [mean (sd): BPD 1.7 (1.6) , No BPD 1.1 (0.3); p < 0.001] and depressive symptoms. However, no difference was observed for MDDs (BPD 32%, no BPD 25%; p = ns)
Kawashima et al. [43]	Cross sectional	Clinical attempters adolescents n = 59 (BPD + BPD traits 27%); adults n = 102	Diagnosis made by DMS-IV and DSM-IV-TR	In comparison to adult attempters, adolescent attempters were more frequently diagnosed with Borderline Personality Disorder (χ^2^(1) = 4.42; p < 0.05), had more school problems and parent loss experience, but they had less financial problems
Yen et al. [44]	Prevalence follow-up = 6 months	Clinical inpatient baseline n = 119; follow up n = 99	Childhood Interview for Borderline Personality Disorders (CI-BPD)	The BPD group had a higher rate of attempts precipitating admission (45% vs. 26%; X^2^ = 4.26, p = 0.04) and were significantly more likely to have a history of suicidal attempts (81% vs. 50%; X^2^ = 10.92, p = 0.001). They found statistically significant correlations between number of BPD criteria endorsed and suicide attempt precipitating admission (r = 0.22, p = 0.018), history of suicide attempts (r = 0.26, p = 0.005), suicidal ideation in the week prior to intake (r = 0.30; p = 0.003) and suicidal ideation in the month prior to intake (r = 0.26; p = 0.008).
Rodgers et al. [45]	Cross sectional	Community sample n = 615	Personality Diagnostic Questionnaire, Fourth Edition (PDQ-4)	Dissociation, borderline traits, and substance use were mediator of the effects of depression on suicidal ideation in the first section of the model, including the defensive latent variable (grouping substance use, borderline features, and dissociative symptoms) was a mediator of the effect of depression on suicidal ideation. Among girls, the model was an excellent fit to the data according to χ^2^, which was non significant (χ^2^ = 9.20, ns); the other indices also reflected good fit (GFI = 0.99; CFI = 1.00, RMSEA = 0.00)
Sharp et al. [46]	Cross sectional	Clinical inpatient n = 156 (BPD n = 30, non BPD n = 126)	(1) Child Interview for DSM-IV Borderline Personality Disorder (CI-BPD); (2) Borderline Features of the Personality Assessment Inventory for Adolescents (PAI)	Results showed that BPD conferred additional risk for suicidal ideation and deliberate self-harm: a diagnosis of MDD or BPD independently increased the odds for thinking about death by nearly 2.5 times [MDD, B = − 0.91; SE = 0.36; Wald statistic (1) = 6.56; p = 0.01, OR = 2.48; BPD, B = − 0.88; SE = .44; Wald statistic (1) = 4.02; df = 1, p < 0.05, OR = 2.42], with addition of BPD to the model robustly improving correct classification of those wishing to die from 29 to 41%. Diagnoses of MDD and BPD independently increased odds for experiencing suicidal ideations by 3.79 and 2.42 times, respectively (MDD, B = − 1.33; SE = 0.36; Wald statistic (1) = 13.98; p < 0.001, OR = 3.79; BPD, B = − 0.89; SE = 0.45; Wald statistic (1) = 3.89; p = 0.05, OR = 2.42)
Yalch et al. [47]	Cross sectional	Clinical inpatients n = 477	Millon Adolescent Clinical Inventory (MACI)	Borderline features (impulsivity β = 0.09 and identity problem β = 0.10) were significantly (r = 0.60, p ≤ 0.01) related to suicide risk even after accounting for symptoms of depression and substance abuse. These findings underscore the clinical value of routinely assessing borderline features among adolescents
Muehlenkamp et al. [48]	Cross sectional	Clinical outpatients n = 441	Structured Clinical Interview for DSM-IV Axis II disorders (SCID II)	ANOVA and logistic regression analyses revealed significant differences across groups, with the BPD symptoms of 'confusion about self' (B = 0.05, Wald= 3.75; Exp(B) = 1.05; p = 0.05) and 'unstable interpersonal relationships' (B = 0.07, Wald= 6.46, Exp(B) = .93; p = 0.01) significantly predicting NSSI and NSSI + Suicide group status
Glenn et al. [49]	Cross sectional	Clinical inpatients n = 97	Borderline Features of the Personality Assessment Inventory for Adolescents (PAI)	Unique associations between borderline personality disorder features and suicide ideation and attempts in adolescents: a hierarchical logistic regression analysis revealed that the BPD significantly distinguished suicide ideators from attempters, over and above demographic and negative emotionality covariates (OR = 1.07, p = 0.03; ∆-2LL = 5.36, p < 0.05)
Somma et al. [50]	Cross sectional	Clinical inpatient n = 85	(1) Personality Inventory for Dsm-5 (PID-5); (2) Structured Clinical Interview for DSM-IV Axis II disorders (SCID II)	With the possible exception of the PID-5 Suspiciousness scale, all other PID-5 scales evidenced adequate internal consistencyreliability (i.e., Cronbach'sαvalues of at least 0.70, most being greater than 0.80). Our data seemed to yield at least partial support for theconstruct validity of the PID-5 scales also in clinical adolescents, at least in terms of patterns of associations with dimensionally assessed DSM-5 “Introduction” section PDs that were also included in theDSM-5AMPD (excluding Antisocial PD because of the participants' minor age). Finally, our data suggested that the clinical usefulness of the PID-5 in adolescent inpatients may extend beyond PDs to profiling adolescentsat risk for life-threatening suicide attempts. In particular, PID-5 Depressivity, Anhedonia, and Submissiveness trait scales were significantlyassociated with adolescents' history of life-threatening suicide attempts, even after controlling for a number of other variables, includingmood disorder diagnosis
Freudenstein et al. [51]	Cross sectional	Clinical inpatients n = 100 (PD 19%)	(1) Child and Adolescent Perfectionism Scale; (2) Narcissistic Personality Inventory (NPI)	The group with high levels of suicidal behavior showed more dependent depression (x = 0.56 and − 0.08, respectively; t = − 2.5; P = 0.014, 2-tailed significance) and socially prescribed perfectionism (x = 1.61 and 1.41, respectively; t = − 1.89; P = 0.031, 1-tailed significance) than the low suicidality group. The suicidal adolescents showed more anaclitic dependence (x = 41.53 and 35.84, respectively; t = − 2.99; P = 0.003, 2-tailed significance) and mature relatedness (x = 38.68 and 34.6, respectively; t = − 2.63; P = 0.01, 2-tailed significance)
Donaldson et al. [52]	Cross sectional	Clinical attempters n = 68	Child and Adolescent Perfectionism Scale (CAPS)	Socially prescribed perfectionism on the CAPS and self-criticism on the DEQ-A were both highly correlated with HSC. Regression analyses indicated that perfectionism was significantly related to hopelessness, but this relationship was attenuated after the effects of depressive cognitions on hopelessness were controlled. Self-criticism was the cognitive variable most strongly associated with hopelessness suggesting that it is a more important focus for cognitive interventions in adolescent suicide attempters than perfectionism
Fennig et al. [53]	Cross sectional	Clinical inpatient n = 404; four groups: 76 male suicide attempters, 103 male nonattempters, 143 female suicide attempters, and 82 female nonattempters.	Aggression (BDI, CSPS, Overt Aggression Scale [OAS], and Multidimensional Anger Inventory); Impulsivity (CSPS and Impulsive Control Scale [ICS]); defense mechanisms (CSPS and LSI)	Logistic regression models revealed that antisocial behavior for man (OR = 3.16, p < 0.0001, 95% CI [1.74, 5.73]) and for women (OR = 1.55, p < .05, 95% CI [0.99, 2.41]) were common predictors of suicide attempt and destructiveness was a predictor in women only (OR = 3.39, p < 0.0001, 95% CI [1.23, 9.32]).
Javdani et al. [54]	Cross sectional	Clinical + community n = 184	Adult Psychopathy Checklist—Revised (PCL)	As predicted, psychopathic traits and depressive symptoms in youth showed differential associations with components of suicidality. Specifically, impulsive traits uniquely contributed to suicide attempts and self-injurious behaviors, above the influence of depression (Wald = 9.24, p < 0.01, OR = 4.08). Indeed, once psychopathic tendencies were entered in the model, depressive symptoms only explained general suicide risk marked by ideation or plans but not behaviors. Further, callous-unemotional traits conferred protection from suicide attempts selectively in girls (Wald = 6.25, p < 0.05, OR = 0.24). For boys, callous/unemotional traits were not significantly related to suicide attempts (Wald = 0.16, p = 0.69, OR = 1.26)
Chabrol and Saint-Martin [55]	Cross sectional	Community n = 288	(1) Borderline Personality Features Scale for Children (BPFS-C); (2) Youth Psychopathic traits Inventory (YPI)	A multiple regression analysis showed that the affective component of psychopathic traits was an independent predictor of suicidal ideation. Our results suggest that clinicians should not assume that the presence of psychopathic traits in adolescent is a protection against suicidal ideation.The variables, as a group, explained 46% of the variance in suicidal ideation. In the second step of the analysis, psychopathic traits were entered: they accounted for an increase of 2% in explained variance. The incremental F ratio was significant (F(5,268) ¼ 3.26, p < 0.01).
Chabrol et al. [56]	Cross sectional	Community n = 615	(1) Borderline Personality Disorder scale of the Personality Diagnostic Questionnaire, Fourth Edition (PDQ-4); (2) Hurting Scale, short version of the Sadistic Attitudes and Behavior Scale (SABS)	The variables, as a group, explained 26.7% of suicidal behavior variance among boys and 30.4% among girls. The contribution of sadistic traits and the interactive relation between sadistic traits and depressive symptoms were evaluated in the second step of the analyses: the addition of these predictors accounted for an additional 3.8% of variance among boys and 9.2% among girls. For both genders, the incremental F ratio (F(2,366) = 10 and F(2,219) = 16.7, respectively) exceeded critical F for the 0.01 level of significance. Sadistic traits appeared to be a significant predictor of suicidality for both genders
Peters et al. [57]	Prevalence follow-up = 6 months	Clinical inpatients n = 103	Childhood Interview for Borderline Personality Disorder (CI‐BPD)	Across the sample, SI intensity, but not lability, was associated with Suicidal Attempts and nonsuicidal self-injury at 6-month follow-up (B = 2.20, 95% CI [1.26–3.85], p < 0.001, X^2^ = 15.78) SI was also associated with borderline personality disorder criteria and a history of sexual abuse (r = 0.22, p < 0.05). Borderline personality disorder criteria was associated with Suicidal Attempts (r = 0.25; p < 0.01)
Peters et al. [57]	Prevalence follow-up = 6 months	Clinical inpatient n = 103	Childhood Interview for Borderline Personality Disorder (CI‐BPD)	Both BPD criteria and a history of sexual abuse were associated with past SAs; in addition, BPD criteria were associated with the SIQ at follow-up and both SAs and NSSI at follow-up In contrast, SI lability, but not SI intensity or initial SI, was significantly associated with greater negative affect intensity and reactivity, as assessed by the AIM
Nakar et al. [58]	Prevalence follow-up = 2 years	Community sample n = 513	15 BPD items from the screening questionnaire for the Structured Clinical Interview for DSM-IV Axis II Personality Disorders (SCID-II-PQ)	Three distinct classes were identified within each of the harmful behaviors. The high-risk trajectories demonstrated elevated initial degree of engagement, followed by a gradual decrease. The observed symptom shift is associated with borderline personality pathology in adolescents, for SIB (F(2,510) = 1229.584, p < 0.001, η^2^ = 0.828), as well as for SB (F(2,510) = 1233.27, p < 0.001, η^2^ = 0.829) and for SM (F(2,510)=1271, 281, p < 0.001, η^2^ = 0.833).

^a^Studies included in the review

#### Longitudinal Studies

In Ayodeji et al. s’ study, the diagnosis of any PD was significantly associated with the severity of suicidal ideation [33].

### Specific Personality Disorder Categories

#### Cross-sectional Studies

Only two autopsy studies investigated the prevalence of specific PD diagnoses in suicidal victims according to the ICD-10 criteria, revealing different distributions between the two studies. In Houson et al. s’ study [30], considering both the categorical diagnosis and the number of criteria, the disorder most frequently diagnosed was the ICD-10 diagnosis of emotionally unstable, which corresponds to the DSM borderline personality disorder (BPD) diagnosis, as shown in Table 1. In Portzky et al. s’ study [31], the most frequent diagnosis was dissocial personality disorder (Table 1). In both studies, other common PD diagnoses were paranoid personality disorder, dependent personality disorder, anankastic personality disorder and anxious personality disorder (Table 1). The overwhelming majority of studies focused on BPD based on DSM criteria, as shown in Table 1. All cross-sectional studies reported a high prevalence of BPD diagnoses in the suicidal population, except for one study in which the percentage of BPD diagnoses amounted to 6% of the clinical sample [37]. The frequency of BPD varied between 56.3 and 90.9% when considering suicidality in terms of both ideation and behaviours [34–36]. Regarding suicide attempts, the percentages ranged from 6 to 27% in clinical inpatient and outpatient samples of fewer than 100 subjects [37–39] and from 48.6 to 62% in samples with more than 100 subjects [40, 41]. Finally, studying suicidal risk in an inpatient sample, Shelby and Yen [42] found 40% of patients with BPD (Table 1). Only two studies investigated suicidal behaviours in BPD samples [43, 44], showing a high incidence of suicide attempts (51.5–76%), as displayed in Table 1. Kawashima and colleagues compared suicide attempters in a mixed adult and adolescent sample, reporting that adolescent attempters were more frequently diagnosed with BPD and were often dealing with the experience of losing a parent or were having problems at school [39]. According to Yen and colleagues, BPD patients had a higher likelihood of being hospitalized for attempting suicide and a higher rate of previous suicide attempts [45]. The relevance of BPD for suicidality in adolescence has also been stressed in studies showing the mediating role of BPD on suicidal ideation and conduct, adding to the impact of mood disorders. Rodgers et al. found that BPD traits represented a significant mediation factor of the effects of depression on suicidal ideation [46]. Moreover, Sharp et al. explored suicide-related behaviours in a psychiatric sample of adolescents at a cross-sectional level, finding that the presence of BPD compared to major depression (MDD) resulted in an increased risk of suicidal ideation [47]. Accordingly, some studies have indicated that specific BPD features can incrementally influence suicidality above the effects of symptoms of depression, substance abuse and other examples of psychopathology [35, 41, 45]. Yalch et al. considered the association between suicide risk and specific BPD features controlling for the effect of depressive symptoms and substance use problems in a sample of clinical inpatients, finding an independent incremental effect of identity disturbance and impulsivity on the variance seen in suicide risk scores [48]. In a clinical outpatient sample, Muehlenkamp et al. reported that the BPD symptoms of ‘confusion about self’ and ‘unstable interpersonal relationships’ allowed them to clearly distinguish between individuals with nonsuicidal self-injuries and those who self-harmed with concomitant suicidal ideation or suicide attempts [49]. Glenn and colleagues demonstrated that in a clinical sample, affective instability provided incremental utility in predicting suicidal behaviour above and beyond negative mood states [50]. Finally, Somma et al. profiled adolescents at risk of life-threatening suicide attempts using the Personality Inventory for DSM-5 (PID-5). The PID-5 traits of depressivity, anhedonia, and submissiveness, which significantly predict BPD diagnoses, were also significantly associated with adolescents’ histories of life-threatening suicide attempts, even after controlling for a number of other variables, including mood disorder diagnoses [51]. Furthermore, depression and BPD seem to play a differential role in the development of suicidal risk: comparing BPD and MDD suicidal patients in terms of the characteristics of their suicidal behaviours, Horesh et al. found that aggression and impulsiveness were in fact positively correlated with suicidal behaviour only among BPD adolescents, whereas hopelessness and depression were positively correlated with suicidal behaviour in both diagnostic groups [43]. Research has also evidenced the relevance of suicidality to other personality dimensions related to diagnoses other than BPD. With the idea that adolescents who attempt suicide may constitute various groups, Cross et al. compared different types of suicide attempters and identified a narcissistic group [23]. Interestingly, this subtype was not characterized by anxiety or mood disorder diagnoses, had minimum substance abuse and conduct problems, was capable of high performance at school and had high adaptive functioning. In contrast, low narcissism scores, measured by the Narcissistic Personality Inventory, have been negatively associated with severe suicidal behaviour [52]. A personality trait that is often associated with a narcissistic personality pathology is perfectionism. Regarding suicidality, the selected studies showed that this personality construct—specifically, socially prescribed perfectionism (SPP)—can be considered an important moderating factor. Indeed, Donaldson and colleagues investigated the relationship between SPP, hopelessness and depressive cognitions in a clinical sample, finding that self-criticism was closely related to hopelessness, which in turn resulted in a greater risk of suicidal behaviour in adolescent attempters [53]. Finally, in a clinical sample, Freudenstein et al. found that adolescent inpatients with high levels of suicidal behaviour were more depressive and more prone to SPP than adolescent inpatients with lower levels of suicidal behaviour [52]. Moreover, some studies have indicated that personality traits that might be considered precursors of antisocial personality disorders (such as aggressiveness, anger, destructive behaviour, impulsivity and conduct disorders) are associated with suicidal behaviour. One study found that all these traits, when present 6 months before admission, were connected to suicidal behaviour. Furthermore, this study found that in male attempters, antisocial behaviours were more present than in female counterparts, although this difference was not significant among non-attempters [54]. The same association between impulsive traits, suicide attempts and self-injurious behaviours was found by Javdani et al. [55]. Interestingly, the authors noted that these traits contributed to self-harming behaviours above the capacity of depression: in this study, that is, depressive symptoms were able to explain only general suicidal ideation, not behaviours. A study by Chabrol and Saint-Martin highlighted that the affective component of psychopathic traits independently predicted suicide; therefore, it would be erroneous for clinicians to assume that psychopathic traits are protective factors against suicide in adolescence [56]. Furthermore, Chabrol et al. found that sadistic traits were connected with suicidality across genders: self-reported sadistic traits explained unique variance in self-reported suicidality in non-clinical adolescents, a result consistent with the psychodynamic hypothesis linking sadism to the self and suicidality [57].

#### Longitudinal Studies

Among the selected articles, eight studies reported follow-up data from 6 months up to over 10 years examining the associations between specific PDs and suicidality. Only six studies focused on short-term outcomes (6 months) following hospitalization due to suicidal risk (severe suicidal ideation or behaviour). Overall, these studies indicated that while suicidal ideation in adolescents with BPD is not stable and decreases rapidly [42], BPD is an independent predictor of suicidal behaviour recurrence at the 6-month follow-up [35, 41, 42]. In Peters et al. s’ study, ideation intensity was the best predictor of suicidal behaviours at this stage and was significantly correlated with BPD traits. The same authors found that BPD criteria were significantly associated with having made a previous suicide attempt, with the presence of suicidal ideation at baseline and with making a subsequent suicide attempt after 6 months [57]. These data were also confirmed by longer longitudinal studies. In a study on the stability and psychopathological associations of BPD characteristics, Greenfield et al. reported that 4 years after recruitment, 94.1% of adolescents who remained suicidal had BPD [36]. Another prospective study with a German cohort of adolescents analysed different trajectories with respect to the evolution of risk behaviours (suicidal conduct, self-harm, drug abuse) for a period of 2 years. The authors identified an incremental relationship between the estimated risk of suicidal behaviour in the high-risk group and BPD criteria [58].

## Discussion

This review of PDs as a risk factor for suicidal ideation and conduct yielded some noteworthy findings. We found evidence of a high prevalence of PDs in adolescent samples with both suicidal ideation and behaviour (Table 1).

The first objective of our review was to demonstrate associations between any PD and suicidal ideation and behaviours. Only one study [32] included in this review focused on the relationship between PD and suicidal ideation, showing how the diagnosis of any PD was significantly associated with the severity of suicidal ideation on a longitudinal basis. Regarding suicidal conduct, our data revealed a higher prevalence of PD diagnosis in the adolescent suicidal population than in community samples [28–31]. In the samples of adolescents with suicidal behaviours, the prevalence amounted to 19.5–22.8% among attempters and 29.6–42.1% among victims. In a recent review by Kongerslev et al., the prevalence of PDs in adolescent community samples and primary care settings generally ranged from 6 to 17% across studies [13]. Johnson and Cohen provided evidence of a differential distribution due to age, with this investigation’s group of 14-year-olds having a prevalence of being diagnosed with any PD of 14.6%, compared to 18.1% among the group of 16-year-olds [59]. This result shows how the prevalence of diagnoses of any PD in the general population is lower than that characterizing the adolescent suicidal clinical population. Based on the data contained in this review, it was not feasible to compare the distribution of personality diagnoses in the adolescent clinical samples, because in all the studies we encountered, it was not possible to ascertain the percentage of suicidal patients included in the samples. With reference to comparisons with adult suicidal populations, the data emerging from this review showed a rate of PDs in the adolescent suicidal population that was comparable and even higher than the distribution of diagnoses of PDs in studies of suicidal adults. In the first review of autopsy studies, Isometsa found a percentage of personality diagnoses that varied between 0 and 57%, with no differentiation between community and clinical samples and no clear discussion as to the reasons for this variability [60]. A meta-analysis by Arsenault-Lapierre et al. confirmed that in adulthood, PDs, along with affective, substance-related and psychotic disorders, accounted for most of the diagnoses among suicidal patients, with a prevalence of PD diagnoses of 16% [9]. As further evidence of the association between PDs and adolescent suicide, the longitudinal study of Grohlt and Ekeberg using a 9-year follow-up after a suicide attempt during adolescence found that the most common disorders in adulthood were depression (46%), PDs (46%) and anxiety disorders (42%) [28].

The second objective of our review was to demonstrate the association between specific personality disorders and/or traits and suicidal ideation and behaviours. Of the studies included in this review, none reported an assessment of the whole range of DSM PD categories. Indeed, the only studies considering the multiplicity of PD diagnoses relied upon the ICD-10 categories [30, 31]. In these studies, diverse personality pathology areas were associated with suicidality, including psychotic, represented by paranoid personality disorder, impulsive-dysregulated, represented by dissocial and emotionally dysregulated personality disorder, and anxious, represented by dependent and anankastic personality disorder. In particular, impulsive-dysregulated personality pathology has been found to be over-represented among suicidal patients [30, 31]. The variants of PDs associated with suicidality certainly deserve more painstaking and systematic exploration. In most of the studies considered in this review, the researchers directly addressed the role of BPD [36–50], probably due the importance that this PD has assumed for adult suicidal patients. Nevertheless, despite its marked incidence in these studies, BPD also showed a wide range of prevalence, with frequencies varying between 56.3 and 90.9% when considering suicidality in terms of both ideation and behaviours [34–36] and between 48.6 and 62% when accounting only for suicide attempts in samples with more than 100 subjects [39, 44]. Three other studies reported percentages lower than 27%, most likely owing to their sample recruitment procedures and smaller sample sizes [37, 38, 45]. Only two studies investigated the rate of suicidal behaviours in BPD samples [41, 42], showing a high incidence of suicide attempts (51.5% and 76%, respectively). Although these studies included only hospitalized patients, it is interesting to compare them with the actual incidence of suicide attempts in the adolescent population: up to 10.6% for girls and 5.4% for boys and up to 3.6% for girls and 1.8% for boys for suicide attempts that required medical attention [61]. The relevance of BPD for suicidality in adolescence has also been stressed by cross-sectional studies showing that BPD mediates the impact of mood disorders on suicidal ideation and conduct [46, 47] and by studies indicating how specific BPD features can incrementally influence suicidality, adding to the effect of depression and substance abuse, two prominent risk factors for adult suicidality [35, 45, 48–51]. Finally, the relationship between BPD diagnosis and suicidality in adolescents also seemed to be confirmed at a longitudinal level, particularly for suicidal behaviours, but not ideation, where the presence of BPD or traits known to be predictors of suicide recurrence. Furthermore, it is important to stress that BPD represents a predictor of future suicide attempts in adolescence independent of other risk factors, thus confirming the utility of such a PD diagnosis for the clinical management of suicidality in this phase of development. One longitudinal study from adolescence through adulthood, not included in this review, evidenced that in a large community sample, BPD symptoms diagnosed in adolescence emerged as the only significant predictor of both proximal and lifetime suicide attempts after controlling for depression severity, early adversity, suicidal ideation and NSSI [62]. Indeed, BPD diagnosis in adolescence remains a stable predictor of future suicide attempts even if the suicide rate originally associated with this diagnosis tends to decrease over time [36]. It should also be noted that adolescent suicidality is a relevant predictor of BPD diagnosis in adulthood [63].

Regarding the third objective, the contributions examined mainly focused on dimensions related to borderline, narcissistic and antisocial traits. The literature has presented evidence that several dimensions related to BPD, namely, affective lability, impulsivity, aggression and identity problems, all seem to exert an important influence on suicidality in adolescence. However, it is unclear whether one of these traits is more effective in predicting suicide [35, 48–51]. This review has also evidenced the relevance of pathological dimensions of personality related to narcissistic personality disorder (NPD) [23, 52, 53], even though no specific reference was made to this DSM diagnosis in the contributions examined. The features or personality traits related to narcissistic pathology associated with suicidality in adolescence comprise a range of symptoms, not all of which are included in the prototypical DSM criteria, and some potentially describe alternative pathological narcissism profiles [23, 53]. In the Collaborative Longitudinal Personality Disorders Study, a 10-year longitudinal research project, NPD was significantly associated with a larger number of suicide attempts, while BPD was significantly associated only with the presence of at least one suicide attempt [64]. Ansell et al. have demonstrated the predictive role of NPD for repetitive suicide attempts, pointing to the role of the peculiar quality of emotional deregulation following narcissistic wounds that lead to increased hostility and aggression [64]. Psychopathological and genetic studies have highlighted a mixed endophenotypical background for both BPD and NPD, characterized by emotional deregulation and hostility that may combine in the two PDs as strong predictors of suicidality [11, 64, 65]. Finally, studies in this review also investigated personality traits associated with antisocial personality organization, such as aggressiveness, anger, destructive behaviour, impulsivity and sadistic traits [55–57], confirming the presence of a suicidal risk in this type of population, as was also observed an adult forensic sample [13].

## Conclusions

Among a vast array of risk factors, PDs are predictive and associated with suicidal ideation and behaviours both at cross-sectional and longitudinal levels in adolescence. From a clinical point of view, these considerations stress the need for personality evaluations in populations at risk of suicidal behaviours. In light of this review, further research clarifying the presence of specific PDs associated with suicidal risk and identifying possible common or single personality dimensions that enhance adolescents’ risk of suicide is necessary. Such a research approach would facilitate more targeted preventive intervention in this area of risk. In general, it should be noted that most of the studies selected in this review reported only data on the rate of PDs in the adolescent suicidal population, whereas a more informative approach could be provided by studies describing the incidence of suicidal ideation and conduct in populations selected on the basis of PD diagnoses. More data should also be collected with follow-up monitoring of suicidal risk in adolescents previously diagnosed with PDs. Finally, many studies excluded from this review have generically pooled together self-harm, ideation and suicide attempts without clearly distinguishing the suicidal nature of self-harm behaviours. Although several studies have evidenced the role of self-harm and NSSI in suicidal risk, it is important to more clearly specify the link between these types of conduct and the various steps of the suicidal process by assessing individuals’ suicidal intent.

## Summary

This work presented a review of research papers examining the role of emerging personality pathology in suicidal ideation and suicidal behaviours in adolescence. The purpose of this contribution was to assess the prevalence and association with suicidal ideations and conduct of (1) overall PD diagnoses or traits, (2) specific PD diagnoses or traits, and (3) personality pathological dimensions.

We therefore searched for studies combining the presence of suicidal ideation or behaviour in clinical or community populations of subjects between 13 and 18 years of age with an emerging personality pathology. We excluded studies that did not distinguish NSSI from suicidality. Out of 2576 generated records, 230 studies were screened in accordance with PRISMA guidelines, with 33 articles considered for this review. The data reviewed revealed percentages ranging from 19.5 to 22.8% of any PD in suicide attempt samples and from 29.6 to 42.1% in autopsy studies of suicidal patients. The overwhelming majority of studies have focused on BPD, with longitudinal studies highlighting the predictive role of BPD for suicidal conduct. This study of pathological personality traits has substantiated that specific BPD features such as affective instability, impulsivity and identity diffusion can incrementally influence suicidality. Furthermore, other personality pathology dimensions, such as aggressiveness, sadism and perfectionism traits, also show a significant mediating role for suicidal risk.

PDs are associated with and help predict suicidal behaviour at both cross-sectional and longitudinal levels. From a clinical point of view, a personality assessment should be mandatory for evaluating suicidal risk, while from a research point of view, more studies integrating categorical and dimensional approaches to the assessment of personality pathology are needed.

## Data Availability

Data sharing is not applicable to this article as no new data were created or analyzed in this study.

## References

[1] World Health Organization (2014). Preventing suicide: a global imperative. Retrieved from https://www.who.int/mental_health/suicide-prevention/world_report_2014/en/

[2] Cha CB, Franz PJM, Guzmán E, GlennKleimanNock CREMMK (2018). Annual research review: suicide among youth—epidemiology, (potential) etiology, and treatment. J Child Psychol Psychiatry.

[3] Centers for Disease Control and Prevention (2020). Retrived from https://www.cdc.gov/violenceprevention/suicide/fastfact.html

[4] Nock MK, Green JG, Hwang I, McLaughlin KA, Sampson NA, Zaslavsky AM, Kessler RC (2013). Prevalence, correlates, and treatment of lifetime suicidal behavior among adolescents: results from the National Comorbidity Survey Replication Adolescent Supplement. JAMA Psychiatry.

[5] Holma KM, Haukka J, Suominen K, Valtonen HM, Mantere O, Melartin TK, Sokero TP, Oquendo MA, Isometsä ET (2014). Differences in incidence of suicide attempts between bipolar I and II disorders and major depressive disorder. Bipolar Disord.

[6] De Crescenzo F, Serra G, Maisto F, Uchida M, Woodworth H, Casini MP, Baldessarini MD, Vicari S (2017). Suicide attempts in juvenile bipolar versus major depressive disorders: systematic review and meta-analysis. J Am Acad Child Adolesc Psychiatry.

[7] Krysinska K, Heller TS, De Leo D (2006). Suicide and deliberate self-harm in personality disorders. Curr Opin Psychiatry.

[8] Giner L, Blasco-Fontecilla H, Perez-Rodriguez MM, Garcia-Nieto R, Giner J, Guija JA, Rico A, Barrero E, Luna MA, de Leon J, Oquendo MA, Baca Garcia E (2013). Personality disorders and health problems distinguish suicide attempters from completers in a direct comparison. J Affect Disord.

[9] Arsenault-Lapierre G, Kim C, Turecki G (2004). Psychiatric diagnoses in 3275 suicides: a meta-analysis. BMC Psychiatry.

[10] Tyrer P, Reed GM, Crawford MJ (2015). Classification, assessment, prevalence, and effect of personality disorder. Lancet.

[11] McGirr A, Renaud J, Bureau A, Seguin M, Lesage A, Turecki G (2008). Impulsive-aggressive behaviours and completed suicide across the life cycle: a predisposition for younger age of suicide. Psychol Med.

[12] Kim CD, Lesage A, Seguin M, Chawky N, Vanier C, Lipp O, Turecki G (2003). Patterns of co-morbidity in male suicide completers. Psychol Med.

[13] Kongerslev MT, Chanen AM, Simonsen E (2015). Personality disorder in childhood and adolescence comes of age: a review of the current evidence and prospects for future research. Scand J Child Adolesc Psychiatry Psychol.

[14] American Psychiatric Association (2013). Diagnostic and statistical manual of mental disorders.

[15] Newton-Howes G, Clark LA, Chanen A (2015). Personality disorder across the life course. Lancet.

[16] Winograd G, Cohen P, Chen H (2008). Adolescent borderline symptoms in the community: prognosis for functioning over 20 years. J Child Psychol Psychiatry.

[17] Caspi A, Houts RM, Belsky DW, Goldman-Mellor SJ, Harrington H, Israel S (2014). The p factor: one general psychopathology factor in the structure of psychiatric disorders?. Clin Psychol Sci.

[18] Sanislow CA, Little TD, Ansell EB, Grilo CM, Daversa M, Markowitz JC (2009). Ten-year stability and latent structure of the DSM–IV schizotypal, borderline, avoidant, and obsessive-compulsive personality disorders. J Abnorm Psychol.

[19] Brown TA, Barlow DH (2005). Dimensional versus categorical classification of mental disorders in the fifth edition of the Diagnostic and statistical manual of mental disorders and beyond: comment on the special section. J Abnorm Psychol.

[20] Kendler KS, Gardner CO, Lichtenstein P (2008). A developmental twin study of symptoms of anxiety and depression: evidence for genetic innovation and attenuation. Psychol Med.

[21] Strickland CM, Hopwood CJ, Bornovalova MA, Rojas EC, Krueger RF, Patrick CJ (2019). Categorical and dimensional conceptions of personality pathology in DSM-5: toward a model-based synthesis. J Pers Disord.

[22] Sevecke K, Schmeck K, Krischer M (2014). The dimensional-categorical hybrid model of personality disorders in DSM-5 from an adolescent psychiatric perspective-criticism and critical outlook. Zeitschrift fur Kinder-und Jugendpsychiatrie und Psychotherapie.

[23] Cross D, Westen D, Bradley B (2011). Personality subtypes of adolescents who attempt suicide. J Nerv Ment Dis.

[24] Turner BJ, Dixon-Gordon KL, Austin SB, Rodriguez MA, Rosenthal MZ, Chapman AL (2015). Non-suicidal self-injury with and without borderline personality disorder: differences in self-injury and diagnostic comorbidity. Psychiatry Res.

[25] Rossouw TI, Fonagy P (2012). Mentalization-based treatment for self-harm in adolescents: a randomized controlled trial. J Am Acad Child Adolesc Psychiatry.

[26] Chanen AM, Jovev M, Djaja D, McDougall E, Yuen HP, Rawlings D, Jackson HJ (2008). Screening for borderline personality disorder in outpatient youth. J Pers Disord.

[27] Groholt B, Ekeberg Ø (2009). Prognosis after adolescent suicide attempt: mental health, psychiatric treatment, and suicide attempts in a nine-year follow-up study. Suicide Life-Threat Behav.

[28] Villar F, Castellano-Tejedor C, Verge M, Sánchez B, Blasco-Blasco T (2018). Predictors of suicide behavior relapse in pediatric population. Span J Psychol.

[29] Houston K, Hawton K, Shepperd R (2001). Suicide in young people aged 15–24: a psychological autopsy study. J Affect Disord.

[30] Portzky G, Audenaert K, van Heeringen K (2005). Suicide among adolescents. Soc Psychiatry Psychiatr Epidemiol.

[31] Tairi T, Milojev P, Zilikis N (2018). Clinical profiles among Greek adolescent suicide attempters: a latent class analysis. Crisis.

[32] Ayodeji E, Green J, Roberts C, Trainor G, Rothwell J, Woodham A, Wood A (2015). The influence of personality disorder on outcome in adolescent self-harm. Br J Psychiatry.

[33] Kuba T, Yakushi T, Fukuhara H, Nakamoto Y, Singeo ST, Tanaka O, Kondo T (2011). Suicide-related events among child and adolescent patients during short-term antidepressant therapy. Psychiatry Clin Neurosci.

[34] Greenfield B, Henry M, Weiss M, Tse SM, Guile JM, Dougherty G, Zhan X, Fombonne E, Lis E, Lapalme-Remis S, Harnden B (2008). Previously suicidal adolescents: predictors of six-month outcome. J Can Acad Child Adolesc Psychiatry.

[35] Greenfield B, Henry M, Lis E, Slatkoff J, Guilé JM, Dougherty G (2015). Correlates, stability and predictors of borderline personality disorder among previously suicidal youth. Eur Child Adolesc Psychiatry.

[36] Fritsch S, Donaldson D, Spirito A, Plummer B (2000). Personality characteristics of adolescent suicide attempters. Child Psychiatry Hum Dev.

[37] Kato K, Mikami K, Nishino R, Akama F, Yamada K, Maehara M (2012). Frequency and clinical features of borderline personality disorder in adolescent suicide attempts in Japan. Asian J Psychiatry.

[38] Yen S, Weinstock LM, Andover MS, Sheets ES, Selby EA, Spirito A (2013). Prospective predictors of adolescent suicidality: 6-month post-hospitalization follow-up. Psychol Med.

[39] Selby EA, Yen S (2014). Six-month trajectory of suicidal ideation in adolescents with borderline personality disorder. Suicide Life-Threat Behav.

[40] Horesh N, Orbach I, Gothelf D, Efrati M, Apter A (2003). Comparison of the suicidal behavior of adolescent inpatients with borderline personality disorder and major depression. J Nerv Ment Dis.

[41] Goodman M, Tomas IA, Temes CM, Fitzmaurice GM, Aguirre BA, Zanarini MC (2017). Suicide attempts and self-injurious behaviours in adolescent and adult patients with borderline personality disorder. Pers Ment Health.

[42] Knafo A, Guilé JM, Breton JJ, Labelle R, Belloncle V, Bodeau N (2015). Coping strategies associated with suicidal behaviour in adolescent inpatients with borderline personality disorder. Can J Psychiatry. Revue canadienne de psychiatrie.

[43] Kawashima Y, Ito T, Narishige R, Saito T, Okubo Y (2012). The characteristics of serious suicide attempters in Japanese adolescents-comparison study between adolescents and adults. BMC Psychiatry.

[44] Yen S, Gagnon K, Spirito A (2013). Borderline personality disorder in suicidal adolescents. Pers Ment Health.

[45] Rodgers RF, van Leeuwen N, Chabrol H, Leichsenring F (2011). An exploration of the role of defensive psychopathology in adolescent suicidal ideation and behavior. Bull Menn Clin.

[46] Sharp C, Green KL, Yaroslavsky I, Venta A, Zanarini MC, Pettit J (2012). The incremental validity of borderline personality disorder relative to major depressive disorder for suicidal ideation and deliberate self-harm in adolescents. J Pers Disord.

[47] Yalch MM, Hopwood CJ, Fehon DC, Grilo CM (2014). The influence of borderline personality features on inpatient adolescent suicide risk. Pers Disord Theory Res Treat.

[48] Muehlenkamp JJ, Ertelt TW, Miller AL, Claes L (2011). Differentiating non-suicidal self-injury in adolescent outpatients: symptoms of borderline personality disorder. J Child Psychol Psychiatry.

[49] Glenn CR, Bagge CL, Osman A (2013). Unique associations between borderline personality disorder features and suicide ideation and attempts in adolescents. J Pers Disord.

[50] Somma A, Fossati A, Terrinoni A, Williams R, Ardizzone I, Fantini F (2016). Reliability and clinical usefulness of the personality inventory for DSM-5 in clinically referred adolescents: a preliminary report in a sample of Italian inpatients. Compr Psychiatry.

[51] Freudenstein O, Valevski A, Apter A, Zohar A, Shoval G, Nahshoni E (2012). Perfectionism, narcissism, and depression in suicidal and nonsuicidal adolescent inpatients. Compr Psychiatry.

[52] Donaldson D, Spirito A, Farnett E (2000). The role of perfectionism and depressive cognitions in understanding the hopelessness experienced by adolescent suicide attempters. Child Psychiatry Hum Dev.

[53] Fennig S, Geva K, Zalzman G, Weitzman A, Fennig S, Apter A (2005). Effect of gender on suicide attempters versus nonattempters in an adolescent inpatient unit. Compr Psychiatry.

[54] Javdani S, Sadeh N, Verona E (2011). Suicidality as a function of impulsivity, callous–unemotional traits, and depressive symptoms in youth. J Abnorm Psychol.

[55] Chabrol H, Saint-Martin C (2009). Psychopathic traits and suicidal ideation in high-school students. Arch Suicide Res.

[56] Chabrol H, van Leeuwen N, Rodgers RF (2011). Exploratory study of the relations between sadistic traits and suicidality in a nonclinical sample of adolescents. Bull Menn Clin.

[57] Peters JR, Mereish EH, Solomon JB, Spirito AS, Yen S (2019). Suicide ideation in adolescents following inpatient hospitalization: examination of intensity and lability over 6 months. Suicide Life-Threat Behav.

[58] Nakar O, Brunner R, Schilling O, Chanen A, Fischer G, Parzer P (2016). Developmental trajectories of self-injurious behavior, suicidal behavior and substance misuse and their association with adolescent borderline personality pathology. J Affect Disord.

[59] Johnson JG, Cohen P, Kasen S, Skodol AE, Oldham JM (2008). Cumulative prevalence of personality disorders between adolescence and adulthood. Acta Psychiatr Scand.

[60] Isometsä ET (2001). Psychological autopsy studies—a review. Eur Psychiatry.

[61] Shain B (2016). Suicide and suicide attempts in adolescents. Pediatrics.

[62] Scott LN, Pilkonis PA, Hipwell AE, Keenan K, Stepp SD (2015). Non-suicidal self-injury and suicidal ideation as predictors of suicide attempts in adolescent girls: a multi-wave prospective study. Compr Psychiatry.

[63] Brière FN, Rohde P, Seeley JR, Klein D, Lewinsohn PM (2015). Adolescent suicide attempts and adult adjustment. Depress Anxiety.

[64] Ansell EB, Wright AG, Markowitz JC, Sanislow CA, Hopwood CJ, Zanarini MC (2015). Personality disorder risk factors for suicide attempts over 10 years of follow-up. Pers Disord Theory Res Treat.

[65] Bornovalova MA, Hicks BM, Iacono WG, McGue M (2009). Stability, change, and heritability of borderline personality disorder traits from adolescence to adulthood: a longitudinal twin study. Dev Psychopathol.

[66] Moher D, Liberati A, Tetzlaff J, Altman DG, The PRISMA Group (2009). Preferred Reporting Items for Systematic Reviews and Meta-Analyses: the PRISMA statement. PLoS Med.

